# A homozygous variant in the *GPIHBP1* gene in a child with severe hypertriglyceridemia and a systematic literature review

**DOI:** 10.3389/fgene.2022.983283

**Published:** 2022-08-16

**Authors:** Ursa Sustar, Urh Groselj, Sabeen Abid Khan, Saeed Shafi, Iqbal Khan, Jernej Kovac, Barbara Jenko Bizjan, Tadej Battelino, Fouzia Sadiq

**Affiliations:** ^1^ Department of Endocrinology, Diabetes and Metabolism, University Children’s Hospital, University Medical Centre Ljubljana, Ljubljana, Slovenia; ^2^ Faculty of Medicine, University of Ljubljana, Ljubljana, Slovenia; ^3^ Division of Cardiovascular Medicine, Department of Medicine, Stanford University, Stanford, CA, United States; ^4^ Department of Paediatrics, Shifa College of Medicine, Shifa Tameer-e-Millat University, Islamabad, Pakistan; ^5^ Department of Anatomy, Shifa Tameer-e-Millat University, Islamabad, Pakistan; ^6^ Department of Vascular Surgery, Shifa International Hospital, Islamabad, Pakistan; ^7^ Department of Vascular Surgery, Shifa Tameer-e-Millat University, Islamabad, Pakistan; ^8^ Clinical Institute for Special Laboratory Diagnostics, University Children’s Hospital, University Medical Centre Ljubljana, Ljubljana, Slovenia; ^9^ Directorate of Research, Shifa Tameer-e-Millat University, Islamabad, Pakistan

**Keywords:** hypertriglyceridemia, triglycerides, glycosylphosphatiliylinositol-anchored high-density lipoprotein-binding protein 1, hyperlipidemia, GPIHBP1

## Abstract

**Background:** Due to nonspecific symptoms, rare dyslipidaemias are frequently misdiagnosed, overlooked, and undertreated, leading to increased risk for severe cardiovascular disease, pancreatitis and/or multiple organ failures before diagnosis. Better guidelines for the recognition and early diagnosis of rare dyslipidaemias are urgently required.

**Methods:** Genomic DNA was isolated from blood samples of a Pakistani paediatric patient with hypertriglyceridemia, and from his parents and siblings. Next-generation sequencing (NGS) was performed, and an expanded dyslipidaemia panel was employed for genetic analysis.

**Results:** The NGS revealed the presence of a homozygous missense pathogenic variant c.230G>A (NM_178172.6) in exon 3 of the *GPIHBP1* (glycosylphosphatidylinositol-anchored high-density lipoprotein-binding protein 1) gene resulting in amino acid change p.Cys77Tyr (NP_835466.2). The patient was 5.5 years old at the time of genetic diagnosis. The maximal total cholesterol and triglyceride levels were measured at the age of 10 months (850.7 mg/dl, 22.0 mmol/L and 5,137 mg/dl, 58.0 mmol/L, respectively). The patient had cholesterol deposits at the hard palate, eruptive xanthomas, lethargy, poor appetite, and mild splenomegaly. Both parents and sister were heterozygous for the familial variant in the *GPIHBP1* gene. Moreover, in the systematic review, we present 62 patients with pathogenic variants in the *GPIHBP1* gene and clinical findings, associated with hyperlipoproteinemia.

**Conclusion:** In a child with severe hypertriglyceridemia, we identified a pathogenic variant in the *GPIHBP1* gene causing hyperlipoproteinemia (type 1D). In cases of severe elevations of plasma cholesterol and/or triglycerides genetic testing for rare dyslipidaemias should be performed as soon as possible for optimal therapy and patient management.

## Introduction

Affecting 15–20% of the population (Parhofer and Laufs, 2019; Basit et al., 2020), hypertriglyceridemia has been associated with an increased risk for pancreatitis (Carrasquilla et al.; Simha, 2020; Álvarez-López et al., 2021). In adults with severe hypertriglyceridemia only up to 2% of the cases could be explained by a monogenic variant in genes involved in triglyceride (TG) metabolism (Hegele et al., 2020). Polygenic variants of smaller effects combined with environmental factors are considered a primary cause of hypertriglyceridemia. Secondary causes include diabetes mellitus, metabolic syndrome, alcohol and commonly used drugs (Simha, 2020).

Hypertriglyceridemia is defined as fasting triglyceride (TG) levels over 2 mmol/L (180 mg/dl), whereas in severe hypertriglyceridemia fasting TG levels exceed 10 mmol/L (885 mg/dl) (Hegele et al., 2020). Patients with the most severe phenotypes start expressing clinical symptoms at a younger age, usually have serum TG levels above 11.3 mmol/L (1000 mg/dl), and in some cases also have abdominal pain related to acute pancreatitis, hepatosplenomegaly, lipemia retinalis, and eruptive xanthomata already in childhood (Gonzaga-Jauregui et al., 2014; Brown et al., 2020; Hegele et al., 2020).

Lipoprotein lipase (LPL) mediates the hydrolysis of triglycerides packed in lipoproteins such as chylomicrons and very-low-density lipoprotein (VLDL) (Wu et al., 2021). Many factors interact with LPL affecting TG metabolism. Dysfunction of LPL and other factors interacting with LPL may lead to hypertriglyceridemia (Liu et al., 2018). Besides *LPL* there are other genes involved in the LPL-mediated lipolysis of chylomicrons and VLDL: *ANGPTL4* (angiopoietin-like 4), *APOC2* (apoprotein C-II), *APOA5* (apolipoprotein A-V), *LMF1* (lipase maturation factor 1), and *GPIHBP1* (glycosylphosphatidylinositol-anchored high-density lipoprotein binding protein 1) (Hegele et al., 2020; Kersten, 2021).

GPIHBP1 binds and transports LPL to the capillary lumen from interstitial space, where it hydrolyses TG and triglyceride-rich lipoproteins (TRLs) (Supplementary Figure S1). In patients with GPIHBP1 deficiency, LPL is mislocalized and intravascular hydrolysis of triglycerides is impaired (Beigneux et al., 2017; Hegele et al., 2020; Wu et al., 2021). The consequence is low plasma levels of LPL, and severe hypertriglyceridemia (Beigneux et al., 2017). The prevalence of type I hyperlipoproteinemia because of a pathogenic variant in the *GPIHBP1* is estimated between 1:500,000 to 1:1,000,000 (Gonzaga-Jauregui et al., 2014).

## Methods

### Study design and family description

The National Medical Ethics Committee approved the study in Slovenia (0120-14/2017/5, and 0120-273/2019/9), and the Ethics Committee approved the study in Pakistan (033-523-2019). The principles of the Declaration of Helsinki were followed. Written consent of the patient’s parents was obtained before inclusion.

### Lipid profile analysis

Serum samples were analyzed for lipids including TC, low-density lipoprotein cholesterol (LDL-C), high-density lipoprotein cholesterol (HDL-C) and TG. TC, TG and LDL-C were measured by a homogenous enzymatic method (Cobas 8000 c502 module, Roche, United States).

Lipid levels were considered as normal if: TC < 4.4 mmoL/L (170 mg/dl), LDL-C <2.8 mmoL/L, (110 mg/dl) HDL-C >1.2 mmoL/L (45 mg/dl), TG < 0.8 mmoL/L (75 mg/dl) for children <9 years of age and <1 mmoL/L (90 mg/dl) if > 9 years of age. Lipid levels were considered as elevated/lowered if: TC > 5.2 mmoL/L (200 mg/dl), LDL-C >3.4 mmoL/L (130 mg/dl), HDL-C <1 mmoL/L (40 mg/dl), and TG > 1.1 mmol/L (100 mg/dl) for children <9 years of age and >1.5 mmoL/L (130 mg/dl) if > 9 years of age. The lipid levels in-between the cut-offs were considered borderline (De Jesus, 2011).

### Genetic analyses

All genetic analyses were performed at the University Children’s Hospital Ljubljana in Slovenia in the same way as Slovenian national genetic testing for the universal familial hypercholesterolemia (FH) screening program in preschool children (Groselj et al., 2018, 2022; Sustar et al., 2022). Genomic DNA was isolated from the patient’s and his family members (mother, father and sister) peripheral blood using a Flexigene kit (Qiagen). xGen^®^ Lockdown^®^ NGS Probes (IDT, United States) for detection of coding and promoter regions for the genes associated with dyslipidemia were used (*ABCA1, ABCG5, ABCG8, ALMS1, APOA1, APOA5, APOB, APOC2, APOC3, APOE, CREB3L3, GPIHBP1, LDLR, LDLRAP1, LIPA, LMF1, LPL,* and *PCSK9*). Samples were sequenced on MiSeq sequencer with MiSeq Reagent Kit (Illumina, United States) following the manufacturer’s protocol including recommendations for quality control parameters. For variant annotation and filtration, the VafAFT tool was applied (Desvignes et al., 2018). The detected variants were classified by the American College of Medical Genetics and Genetics and the Association for Molecular Pathology (ACMG-AMP) (Richards et al., 2015) classification criteria as (likely) benign, variants of uncertain significance (VUS) and (likely) pathogenic. The pathogenic variant in the *GPIHBP1* gene was reconfirmed by targeted Sanger DNA sequencing.

### Systematic literature review

We gathered all accessible scientific case report publications for the systematic review of pathogenic variants in the *GPIHBP1* gene. The systematic review was registered at PROSPERO (CRD42022336232). An electronic search was performed using the keyword “GPIHBP1” in the PubMed database on 11 June 2022. Moreover, we searched for the articles related to pathogenic variants also through the search in the Human Gene Mutation Database (Stenson et al., 2003) and the Franklin by Genoox tool based on the pathogenic variant, confirmed in our patient “NM 178172.6:c.230G>A” (scope: “Gene”). By going through all of the abstracts and titles found, we included all articles meeting the following requirements: 1) articles in English published after 2002, 2) articles containing human data, 3) only articles or data from articles describing pathogenic variants in the *GPIHBP*1 gene, 4) reported patients were homozygous or compound heterozygous for pathogenic variants, and 5) clinical data on patients was provided.

## Results

**FIGURE 1:**
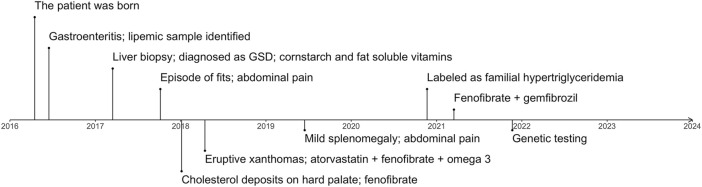
The timeline represents important complications and milestones in diagnosing and managing the patient. GSD, glycogen storage disease.

The proband was a 5.5-year-old boy. He was born at a full-term birth weight of 3.7 kg with no known antenatal issues. The timeline of the diagnosing, treatment and management of the patient is represented in Figure 1. The parents of the patient are in a consanguineous marriage and are first-degree relatives. The patient’s mother has had an early abortion and has a 2-year-old healthy daughter. The maternal grandfather of the patient had a heart attack at 28 years of age. No family history of pancreatitis is known. The lipid profile of the proband and his family members is represented in Supplementary Figure S2.

**FIGURE 2:**
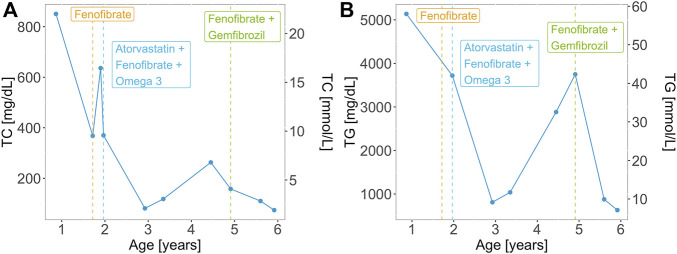
**(A)** Total cholesterol (TC) and **(B)** triglyceride (TG) levels over time for the patient with the homozygous GPIHBP1 pathogenic variant. Vertical lines represent the initiation and/or modification of the treatment.

At 2 months of age, the patient had an episode of gastroenteritis needing intravenous hydration. At that time mother was first notified of “pink blood” (lipemic sample). Repeated sampling confirmed the same finding. The child was later taken to a local hospital where he was seen by a paediatric gastroenterologist who advised liver biopsy. The child had an initial liver biopsy done at 10 months of age, which showed a severely autolytic sample with Periodic acid–Schiff (PAS) stain positive in preserved areas suggestive of glycogen storage disease (GSD). At the same time, other laboratory measurements were elevated: total serum cholesterol of 850.7 mg/dl (22.0 mmol/L) and serum triglycerides of 5,137 mg/dl (58.0 mmol/L). However, fasting glucose was raised significantly (29.5 mmol/L) along with total bilirubin levels (110.0 umol/L) and ALT 550 U/L. The TC and TG levels of the patient over time are represented in Figure 2. On examination, he had pallor and a soft abdomen with no hepatomegaly. The patient was labelled as a case of GSD. Based on that, the patient was advised to start taking cornstarch, fenofibrate, allopurinol, and vitamins A, D and E.

Aged 1 year and 5 months the patient presented to the Paediatric outpatient department in Shifa Hospital for the first time with complaints of one episode of vague seizure-like activity and abdominal pain for 3 months. At age 1 year and 8 months, the patient developed hoarseness of voice. He was noted to have 2–3 yellow plaques on the hard palate. At this visit, his TC level was 368.0 mg/dl (9.5 mmol/L). Two months later he had eruptive xanthomas, a TC levels of 636.0 mg/dl (16.4 mmol/L). Aged 2 his TC levels were 370 mg/dl (9.6 mmol/L) and his TG levels were 3,720 mg/dl (42.0 mmol/L). Along with fenofibrate and omega 3 the patient was prescribed atorvastatin (5 mg) with increasing dosage (10 mg) upon a follow-up visit. Treatment of the patient over time is represented in Figure 2. The patient presented for follow-ups with lab results that repeatedly showed TG and total serum cholesterol above normal limits. He never had any documented hypoglycemia, drowsiness or seizure activity. He didn’t have hepatomegaly.

Two years following the initial presentation, with regular follow-ups in between, the boy presented to the outpatient department with pain in the abdomen, lethargy and poor appetite. There was mild splenomegaly and upon repetition of laboratory tests, ALT (29 U/L) and serum lipase levels were normal, TC level was 81.0 mg/dl (2.1 mmol/L), LDL-C 18.0 mg/dl (0.5 mmol/L), HDL 8 mg/dl (0.2 mmol/L), triglycerides were 1,068 mg/dl (12.1 mmol/L) and 2,883 mg/dl (32.6 mmol/L) subsequently, despite receiving the treatment with atorvastatin, fenofibrate 50 mg once daily and omega 3 capsules twice a day.

The diagnosis was reviewed at age 4.5 years due to persistently high serum TG and relabeled as a case of primary/familial hypertriglyceridemia based on the lipid profile. The patient was advised to stop using cornstarch, allopurinol and atorvastatin and continue fenofibrate. On follow-up, the patient, weighing 14 kg at 4 years and 11 months of age, had his TC within normal range, however, TGs were still deranged (3749 mg/dl, 42.3 mmol/L). Response to fenofibrate (67 mg twice a day) was inadequate with persistent high TGs (>1000 mg/dl; 11.3 mmol/L), therefore gemfibrozil (300 mg twice a day) was added to the treatment regime. Aged 5 years and 7 months the triglycerides were 878.0 mg/dl (9.9 mmol/L), TC 110.0 mg/dl (2.8 mmol/L), LDL-C 10 mg/dl (0.3 mmol/L), HDL-C 0.2 mmol/L (8.0 mg/dl). His weight and height were at the 5th centile for his age and gender. At age 5 years and 11 months his lipid profile levels were: TC: 74 mg/dl (1.9 mmol/L), LDL-C: 10 mg/dl (0.3 mmol/L), HDL-C: 8 mg/dl (0.2 mmol/L) and TG: 630 mg/dl (7.1 mmol/L). At that point, he had no xanthomas.

At age 5.5 genetic testing was performed and we identified a homozygous variant c.230G>A (NM_178172.6) in exon 3 of the *GPIHBP1* gene (NG_034256.1) leading to a protein change p.Cys77Tyr (NP_835466.2). The patient’s family members (father, mother and sister) were heterozygous for the c.230G>A variant (Supplementary Figures S2, S3). The variant has already been reported in the ClinVar (VCV000917845.1) (Landrum et al., 2018) as likely pathogenic in association with hyperlipoproteinemia (type ID) phenotype. The variant was classified as pathogenic by the *in silico* prediction tools (Revel, MetaLR, MetaSVM) (Ioannidis et al., 2016). The frequency of the variant in the gnomAD population databases (Karczewski et al., 2020) is extremely low. Following the American College of Medical Genetics and Genetics and the Association for Molecular Pathology (ACMG-AMP) criteria (Richards et al., 2015) the variant was classified as likely pathogenic.

In Table 1 we reviewed the literature on the pathogenic variants in the *GPIHBP1* gene containing additional clinical information about the patients. Fifty-four patients were homozygous while seven were compound heterozygous for a pathogenic variant in the *GPIHBP1* gene. One patient was heterozygous for variants in *GPIHBP1* and *APOC2* genes. We presented 32 unique variants in the *GPIHBP1* gene. 5 pathogenic variants are located in exon 1, 3 in exon 2, 10 in exon 3 and 10 in exon 4. 4 variants represent major deletion of a whole exon/multiple exons/whole *GPIHBP1* gene.

**TABLE 1:** Review of pathogenic variants in *GPIHBP1* gene from the literature.

Reference	Nationality	Gender	Age	HGVS Transcript and Protein Change	Zygosity	TG (mg/dL)	TG (mmol/L)	EX	HSM	AP	C
(Ariza et al., 2016)	Ecuadorean	F	25	NM_178172.6:c.3G>T, NP_835466.2:p.(Met1?)	HOM	3,82	43.1	No	No	Yes	Yes
(Hegele et al., 2018)				NM_178172.6:c.17C>A, NP_835466.2:p.(Ala6Asp)	HOM						
(Paquette et al., 2018)	Vietnamese	F	33	NM_178172.6:c.40_41insGCGG, NP_835466.2:p.(Phe14CysfsTer25)	HOM	5,973	67.4	No	No	Yes	No
(Yamamoto et al., 2013)	Japanese	F	54	NM_178172.6:c.202T>C, NP_835466.2:p.(Cys68Arg)	HOM	2,64		No	No	Yes	Yes
(Liu et al., 2022)	Chinese	F	29 days	NM_178172.6:c.45_48dupGCGG, NP_835466.2:p.(Pro17AlafsTer22)	HOM	2,255	25.46		No		No
(Lin et al., 2020)	Chinese	F	35	NM_178172.6:c.48_49insGCGG, NP_835466.2:p.(Pro17AlafsTer22)	HOM	1,514	17.09	No	Yes	Yes	
(Ahmad and Wilson, 2014)	Caucasian	F	2 mth	NM_178172.6:c.85_88GAGGdel, NP_835466.2:p.(Glu29ThrfsTer50)	CHET	2,663	30.1	Yes	No	Yes	No
				NM_178172.6:c.267C>A, NP_835466.2:p.(Cys89Ter)							
(Buonuomo et al., 2015)	Italian		3 days	NM_178172.6:c.154_162AACAGGCTCdelTCTTins, NP_835466.2:p.(Asn52SerfsTer253)	CHET	1,667	18.8			No	
				NM_178172.6:c.319T>C, NP_835466.2:p.(Ser107Pro)							
(Wang and Hegele, 2007)		F	47	NM_178172.6:c.166G>C, NP_835466.2:p.(Gly56Arg)	HOM	7,094	80.1			Yes	No
(Wang and Hegele, 2007)		M	52	NM_178172.6:c.166G>C, NP_835466.2:p.(Gly56Arg)	HOM		48.0			Yes	
(Lima et al., 2021)	Brazilian	F	30	NM_178172.6:c.182-1G>T, NP_835466.2:p.?	HOM	2,498	28.2	No	No	Yes	No
(Lima et al., 2021)	Brazilian	F	11	NM_178172.6:c.182-1G>T, NP_835466.2:p.?	HOM	1,248	14.1	No	No	No	No
(Lima et al., 2021)	Brazilian	M	15	NM_178172.6:c.182-1G>T, NP_835466.2:p.?	HOM	2,204	24.9	Yes	No	No	No
(Lima et al., 2021)	Brazilian	F	48	NM_178172.6:c.182-1G>T, NP_835466.2:p.?	HOM	2,84	32.1	Yes	No	Yes	Yes
(Lima et al., 2021)	Brazilian	F	42	NM_178172.6:c.182-1G>T, NP_835466.2:p.?	HOM	6,381	72.0	Yes	No	Yes	Yes
(Lima et al., 2021)	Brazilian	F	37	NM_178172.6:c.182-1G>T, NP_835466.2:p.?	HOM	1,975	22.3	No	No	Yes	No
(Lima et al., 2021)	Brazilian	F	1	NM_178172.6:c.182-1G>T, NP_835466.2:p.?	HOM	20,6	232.6	No	No	No	No
(Lima et al., 2021)	Brazilian	F	1	NM_178172.6:c.182-1G>T, NP_835466.2:p.?	HOM	4,142	46.8	Yes	No	No	Yes
(Lima et al., 2021)	Brazilian	F	30	NM_178172.6:c.182-1G>T, NP_835466.2:p.?	HOM	885	10.0	No	No	No	Yes
(Lima et al., 2021)	Brazilian	M	27	NM_178172.6:c.182-1G>T, NP_835466.2:p.?	HOM	924	10.4	No	No	No	Yes
(Lima et al., 2021)	Brazilian	F	0.5	NM_178172.6:c.182-1G>T, NP_835466.2:p.?	HOM	18	203.2	No	No	No	No
(Lima et al., 2021)	Brazilian	M	0.6	NM_178172.6:c.182-1G>T, NP_835466.2:p.?	HOM	40,141	453.2	No	No	No	Yes
(Surendran et al., 2012)	Dutch			NM_178172.6:c.194G>A, NP_835466.2:p.(Cys65Tyr)	HOM						
(Franssen et al., 2010)	UAE	M	3	NM_178172.6:c.194G>A, NP_835466.2:p.(Cys65Tyr)	HOM	4,005	45.2	No	No	Yes	
(Olivecrona et al., 2010)	Swedish	M	10	NM_178172.6:c.194G>C, NP_835466.2:p.(Cys65Ser)	CHET	1,727	19.5	No	Yes		No
				NM_178172.6:c.202T>G, NP_835466.2:p.(Cys68Gly)							
(Iacocca et al., 2019)	Swedish	F	9 mth	NM_178172.6:c.194G>C, NP_835466.2:p.(Cys65Ser)	CHET	5,049	57.0	No	Yes	Yes	No
				NM_178172.6:c.202T>G, NP_835466.2:p.(Cys68Gly)							
(Iacocca et al., 2019)	Swedish	F	16 mth	NM_178172.6:c.194G>C, NP_835466.2:p.(Cys65Ser)	CHET	4,296	48.5	No	Yes	Yes	No
				NM_178172.6:c.202T>G, NP_835466.2:p.(Cys68Gly)							
(Coca-Prieto et al., 2011)	Spanish	F	30	NM_178172.6:c.203G>A, NP_835466.2:p.(Cys68Tyr)	HOM	>1,000	>11.3	No	No	Yes	
(Chokshi et al., 2014)	Salvadorean	F	24	NM_178172.6:c.203G>A, NP_835466.2:p.(Cys68Tyr)	HOM			Yes	Yes	Yes	No
(Rios et al., 2012)	Salvadorean	F	36	NM_178172.6:c.203G>A, NP_835466.2:p.(Cys68Tyr)	HOM	6,48	73.2	Yes	No	Yes	No
(Ariza et al., 2016)	Pakistani	M	39	NM_178172.6:c.239C>A, NP_835466.2:p.(Thr80Lys)	HOM	4,489	50.7	No	No		
(Rabacchi et al., 2016)			42	NM_178172.6:c.247T>C, NP_835466.2:p.(Cys83Arg)	HOM					Yes	
(Rabacchi et al., 2016)		M	40	NM_178172.6:c.247T>C, NP_835466.2:p.(Cys83Arg)	HOM					No	
(Charrière et al., 2011)		M	6 mth	NM_178172.6:c.266G>T, NP_835466.2:p.(Cys89Phe)	CHET	1,736	19.6			Yes	
				ex1_3 del							
(Rabacchi et al., 2016)		F	55	NM_178172.6:c.267C>A, NP_835466.2:p.(Cys89Ter)	HOM				Yes	Yes	
(Plengpanich et al., 2014)	Thai	F	46	NM_178172.6:c.320C>G, NP_835466.2:p.(Ser107Cys)	HOM	3,164	35.7	No		No	
(Plengpanich et al., 2014)	Thai	M	64	NM_178172.6:c.320C>G, NP_835466.2:p.(Ser107Cys)	HOM	842	9.5			No	
(Plengpanich et al., 2014)	Thai	M	43	NM_178172.6:c.320C>G, NP_835466.2:p.(Ser107Cys)	HOM	673	7.6			No	
(Chyzhyk et al., 2019)	Middle East	F	6 weeks	NM_178172.6:c.323C>G, NP_835466.2:p.(Thr108Arg)	HOM	3,15	35.6			No	No
(Chyzhyk et al., 2019)	Middle East	F	2	NM_178172.6:c.323C>G, NP_835466.2:p.(Thr108Arg)	HOM	1,838	20.8			Yes	No
(Surendran et al., 2012)	Caucasian	M	1	NM_178172.6:c.323C>G, NP_835466.2:p.(Thr108Arg)	HOM					Yes	
(Gonzaga-Jauregui et al., 2014)	Spanish	F	5 weeks	NM_178172.6:c.331A>C, NP_835466.2:p.(Thr111Pro)	CHET	12,046	136.0	No		Yes	No
				NM_178172.6:c.413_429del, NP_835466.2:p.(Pro140SerfsTer161)							
(Beigneux et al., 2009a)	Colombian	M	33	NM_178172.6:c.344A>C, NP_835466.2:p.(Gln115Pro)	HOM	3,366	38.0	No	Yes	No	
(Surendran et al., 2012)	Dutch			NM_178172.6:c.344A>C, NP_835466.2:p.(Gln115Pro)	HOM						
(Hegele et al., 2018)				NM_178172.6:c.394C>T, NP_835466.2:p.(Gln132Ter)	HOM						
(Jung et al., 2017)	Algerian	M	1 mth	NM_178172.6:c.476delG, NP_835466.2:p.(Gly159AlafsTer94)	HOM	>5,000		No	No	No	
(Ariza et al., 2018)	Spanish			NM_178172.6:c.502delC, NP_835466.2:p.(Leu168SerfsTer85)	HOM						No
(Charrière et al., 2011)		M	35	NM_178172.6:c.523G>C, NP_835466.2:p.(Gly175Arg)	HOM	2,303	26.0			Yes	
(Rios et al., 2012)	Algerian	M	26	NM_178172.6:c.523G>C, NP_835466.2:p.(Gly175Arg)	HOM	2,303					No
(Chyzhyk et al., 2019)		M	43	NM_178172.6:c.523G>C, NP_835466.2:p.(Gly175Arg)	HET	>5,000	>56.4			Yes	
				NM_000483.5:c.2-4G>C, NP_000474.2:p.?							
(Berge et al., 2014)	Pakistani	F	22	ex3 and 4 deletion	HOM	5,314	60.0			Yes	Yes
(Berge et al., 2014)	Pakistani	M	37	ex3 and 4 deletion	HOM	8,857	100.0			Yes	Yes
(Berge et al., 2014)	Pakistani	M	40	ex3 and 4 deletion	HOM	2,073	23.4			No	Yes
(Berge et al., 2014)	Pakistani	F	37	ex3 and 4 deletion	HOM	2,028	22.9			Yes	Yes
(Iacocca et al., 2019)		M	22	ex3 and 4 deletion	HOM	2,737	30.9			No	
(Iacocca et al., 2019)		M	39	ex3 and 4 deletion	HOM	1,329	15.0			No	
(Iacocca et al., 2019)		M	50	ex3 and 4 deletion	HOM	957	10.8			Yes	
(Rios et al., 2012)	Indian	M	2 mth	17.5 kbp deletion (included GPIHBP1)	HOM	37,284	421.0	No	No	No	No
(Iacocca et al., 2019)	Indian	F	44	17.5 kbp deletion (included GPIHBP1)	HOM					Yes	No
(Patni et al., 2016)	Indian	M	10	Entire gene deletion (54,623 bp)	HOM	2,274	25.7	No	Yes	No	No
(Chokshi et al., 2014)	Indian	M	2 mth	Entire gene deletion	HOM	>20,000	>225.8	No	No	Yes	No
(Iacocca et al., 2019)		M	37	Entire gene deletion	HOM	3,082	34.8			Yes	

HO, homozygous; CHE, compound heterozygous; HE, heterozygous; EX, eruptive xanthoma; HSM, hepatosplenomegaly; AP, acute pancreatitis; C, consanguinity.

## Discussion

We presented a patient with severe hypertriglyceridemia as a consequence of a homozygous pathogenic variant in the *GPIHPB1* gene. Additionally, we reviewed the literature on described cases with pathogenic variants in the *GPIHBP1* gene.

Elevated TG levels have been linked to cardiovascular disease (CVD) and pancreatitis (Carrasquilla et al., 2021). For severe hypertriglyceridemia, pathogenic variants in genes *LPL*, *APOA5*, *APOC2*, *GPIHBP1,* and *LMF1*, associated with hyperlipidemia should be considered (Goldberg and Chait, 2020). *GPIHBP1* is located on chromosome 8q24.3 and is composed of 4 exons (Liu et al., 2018). *GPIHBP1* is expressed mostly in the capillary endothelial cells of the heart, brown adipose tissue and skeletal muscle, involved in energy and lipid metabolism (Cruz-Bautista et al., 2021). GPIHBP1 acts as an important partner of the LPL in plasma triglyceride metabolism (Supplementary Figure S1). The loss-of-function pathogenic variant in *GPIHBP1* impairs LPL activity and its lipolytic processing of chylomicrons and very-low-density lipoproteins (Voss et al., 2011; Gin et al., 2012). LPL unfolds spontaneously and remains within the interstitial spaces. Due to the loss of its catalytic activity, triglycerides are not hydrolysed resulting into severe hypertriglyceridemia (Arora et al., 2019; Miyashita et al., 2020) (Supplementary Figure S1).

GPIHBP1 is a protein composed of 184 amino acids from the lymphocyte antigen (Ly6) family and consists of a N-terminal signal peptide region, an amino-terminal acidic domain, a Ly6 domain of ten disulfide-bonded cysteines and a highly hydrophobic carboxyl-terminal motif that is replaced within the endoplasmic reticulum by a glycosylphospatidylinostiol (GPI) anchor for tethering to the cell membrane. GPIHBP1 is distinguishable from the other Ly6 family members by the presence of a high acidic domain composed of 21 aspartates or glutamates located 12 amino acids prior to the Ly6 motif (Beigneux A. R. et al., 2009; Liu et al., 2018). Both, a high acidic and Ly6 domain are important for the GPIHBP1 and LPL interaction. Genetic modifications encoding for one of these two domains result in chylomicronemia. Most missense variants break down the disulfide bond of the Ly6 domain, resulting in the production of GPIHBP1 dimers and multimers, which are incapable of binding LPL (Beigneux et al., 2015; Fong et al., 2016).

**FIGURE 3:**
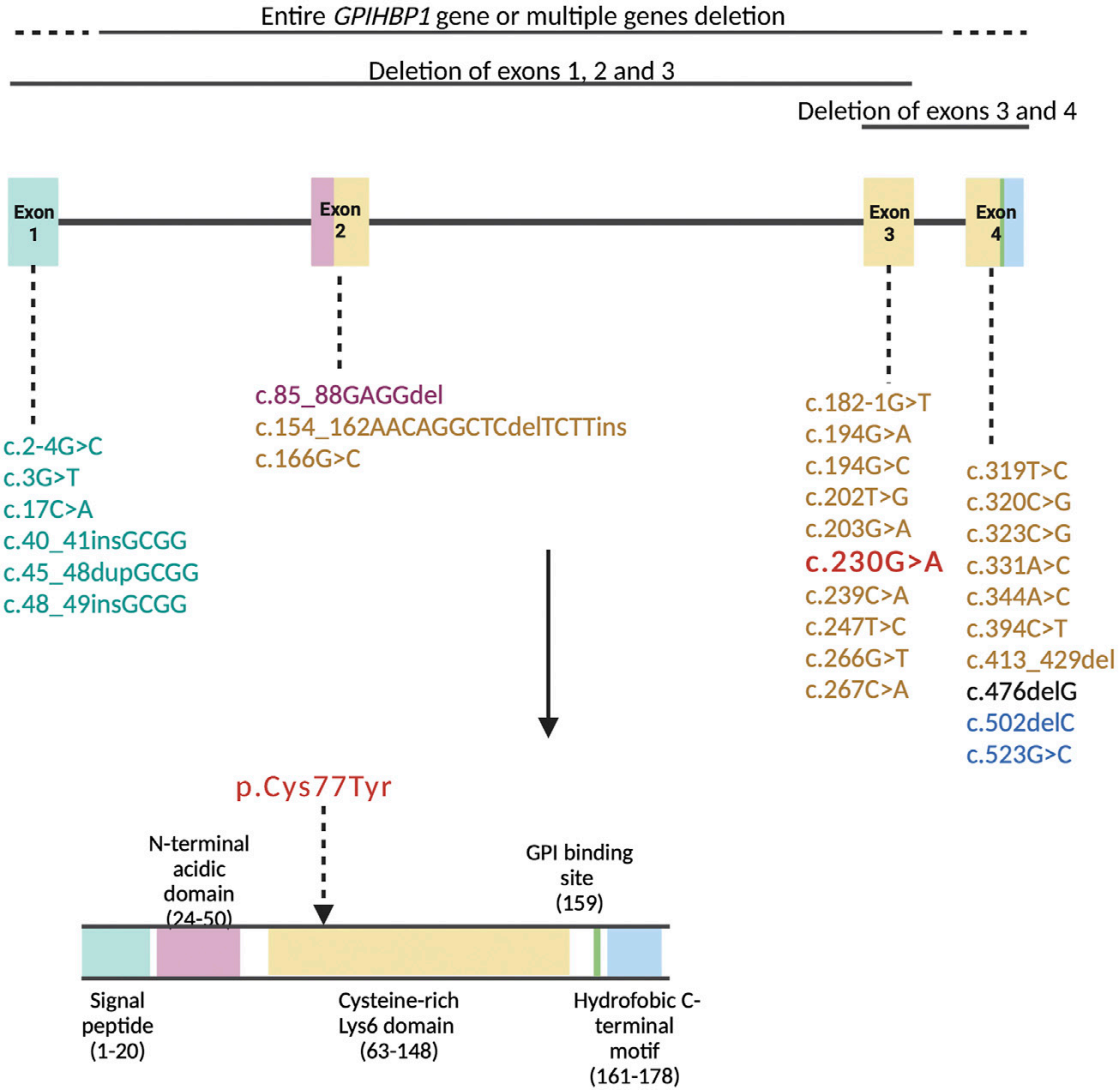
Pathogenic variants in the *GPIHBP1* gene based on the literature review represented in Table 1 are organized by exon numbers. The colour of the exon denotes the protein domain affected by the pathogenic variation. The variant of our patient (c.230G > A) is in bold red colour.

Amino acids at positions 24-50 (exon 2) compose the acidic domain and amino acids at positions 63-148 (exons 3 and 4) compose the Ly6 domain of the GPIHBP1 protein (Figure 3). Amino acids at positions 27-50 (exon 2) are important for LPL transport to the lumenal surface of endothelial cells. Amino acids at positions 103-109 (exon 4) are important for interaction with LPL (UniProt, Q8IV16). The majority of pathogenic variants disrupt the Ly6 domain’s folding, resulting in multimerized and defective GPIHBP1 molecules on the cell surface (Holmes and Cox, 2012; Mysling et al., 2016).

Patients with GPIHBP1 deficiency express a similar phenotype as patients with LPL deficiency, such as severe chylomicronemia (plasma TG levels above 1,500 mg/dl (16.9 mmol/L) presented already in childhood and high risk of pancreatitis (Miyashita et al., 2020). Highest reported TG and TC levels for the patient at age 10 months were 5,137 mg/dl (58.0 mmol/L) and 850.7 mg/dl (22.0 mmol/L) respectively. Because hyperbilirubinemia was detected during this examination, extreme TC levels could be the consequence of biliary congestion. Hyperbilirubinemia was not detected on further examinations, yet increased TC and TG levels remained.

Moreover, as the recessive manner of inheritance of the disorder, the parents and the younger sister of the patient did not express the extreme phenotype associated with the disorder, although the father has elevated TC and TG levels (Supplementary Figure S1), similarly, as reported by Wang and Hegele, 2007 for heterozygous family members of a proband with a homozygous variant in the *GPIHBP1* gene. Likewise, our patient’s father may have an extra genetic variant that contributes to his phenotype but is not present in the patient’s mother or sister.

The variant p.Cys77Tyr of our patient is located in exon 3, encoding for the Ly6 domain of the GPIHBP1 protein (Figure 3). Cysteine residues of Ly6 domain are essential for the 3-fingered structural motif formation. Interfering with any of the disulfide links is expected to cause significant structural changes in the protein (Rabacchi et al., 2016). Beigneux et al. (2009b) report that cells expressing the cysteine mutants in *GPIHBP1* are unable to bind and transport LPL from the subendothelial space to the endoluminal surface of the endothelial cells. Furthermore, several genetic variants involving cysteine alterations to another amino acid have previously been documented. Eight patients with homozygous and four with compound heterozygous variants p.Cys65Tyr, p.Cys68Gly, p.Cys83Arg, p.Cys83Arg, p.Cys89Phe and p.Cys89Ter (Olivecrona et al., 2010; Charrière et al., 2011; Iacocca et al., 2019) with severe elevation in TG (1,000–6,480 mg/dl, 11.3–73.2 mmol/L) and episodes of acute pancreatitis have previously been described. Moreover, Lima et al. (2021) present a series of twelve cases with an intronic variant c.182-1G>C, resulting in the skipping of cysteine-rich exon 3. These patients likewise had extremely elevated TG levels (885–40,141 mg/dl, 10–453.2 mmol/L), but interestingly only four suffered from acute pancreatitis, none of them had hepatosplenomegaly and four of them had eruptive xanthomas. Additionally, twelve patients with deletion of exon 3 and 4 or entire gene deletion had TG values between 957 and 37,284 mg/dl (10.8 and 421.0 mmol/L) and seven of them were affected by acute pancreatitis (Rios et al., 2012; Berge et al., 2014; Chokshi et al., 2014; Patni et al., 2016; Iacocca et al., 2019). Table 1 contains additional information about these patients.

European Atherosclerosis Society provided practical clinical guidelines for rare dyslipidaemia management for patients with extreme LDL-C, TG and HDL-C levels (Hegele et al., 2020). Early detection of rare dyslipidemias in a pre-clinical stage is possible with an effective FH screening program capable of detecting other dyslipidemia than FH (Groselj et al., 2018, 2022; Marusic et al., 2020; Sustar et al., 2022) or with a gene panel applied as a part of a newborn screening program (Remec et al., 2021). It is important to implement a worldwide registry for rare dyslipidemias, comparable to what already exists for FH/homozygous FH (Tromp et al., 2022; Vallejo-Vaz et al., 2018, 2021).

Statin therapy substantially reduced the CVD risk in patients with high LDL-C levels (Silverman et al., 2016). Nevertheless, other factors, such as triglycerides or triglyceride-rich lipoproteins (TRLs), contribute to the CVD risk with low-grade inflammation, as a part of atherosclerosis (Nordestgaard, 2016). The goal of the treatment is to reduce plasma TG levels to less than 500–1,000 mg/dl to prevent acute pancreatitis (Okazaki et al., 2021). To prevent abdominal pain and acute pancreatitis, patients should be on a low-fat diet of total dietary fat intake of <10–15% of daily calories (<15–20 g per day) and treated with common lipid-lowering drugs (fibrates, omega-3 fatty acids, statins) (Esan and Wierzbicki, 2020; Navarro Hermoso and Valdivielso, 2021). Volanesorsen, an antisense oligonucleotide inhibitor of apoC3 is a promising medicine for the reduction of TG levels by 70–80% (Esan and Wierzbicki, 2020). Furthermore, gemfibrozil is a useful medication for the reduction of TGs in patients with very high TG serum levels. The mechanism of action of gemfibrozil is based on the activation of nuclear transcription factors for up-regulation of LPL transcription and down-regulation of the LPL inhibitor apo C-III, resulting in a decrease in triglyceride levels and an increase in HDL. Moreover, gemfibrozil reduces hepatic triglyceride synthesis by inhibiting peripheral lipolysis and decreasing hepatic removal of free fatty acids. It inhibits the synthesis and increases the clearance of very low-density lipoprotein (VLDL) (Goldberg and Hegele, 2012). Our patient was managed with statins (atorvastatin) and later with a combination of fibrates (fenofibrate and gemfibrozil). Combination therapy with fenofibrate and gemfibrozil has helped in lowering TG levels (<1000 mg/dl) which was not achieved by monotherapy with fenofibrate. Statins were stopped when the diagnosis of GSD was dismissed, and the patient was treated as a case of primary HTG.

In conclusion, genetic testing for rare dyslipidaemias should be considered early in cases of severe elevations of plasma cholesterol and/or triglycerides, to enable adequate and precise management of the patient. Besides *LPL*, there are other genes involved in primary hypertriglyceridemia phenotypes (most notably, *APOC2, APOA5, LMF1,* and *GPIHBP1*). Our paediatric patient had a homozygous pathogenic *GPIHBP1* variant, causing severe hypertriglyceridemia, cholesterol deposits at the hard palate, eruptive xanthomas, lethargy, poor appetite, and mild splenomegaly. Combination treatment with fenofibrate and gemfibrozil was shown to help reduce TG levels.

## Data Availability

The datasets for this article are not publicly available due to concerns regarding participant/patient anonymity. Requests to access the datasets should be directed to the corresponding author.

## References

[B1] Ahmad Z. Wilson D. P. (2014). Familial chylomicronemia syndrome and response to medium-chain triglyceride therapy in an infant with novel mutations in GPIHBP1. J. Clin. Lipidol. 8, 635–639. 10.1016/j.jacl.2014.08.010 25499947

[B2] Álvarez-López H. Ruiz-Gastélum E. Díaz-Aragón A. (2021). Tratamiento actual de la hipertrigliceridemia. Cardiovasc. Metab. Sci. 32, 242–246. 10.35366/100805

[B3] Ariza M. J. Martínez-Hernández P. L. Ibarretxe D. Rabacchi C. Rioja J. Grande-Aragón C. (2016). Novel mutations in the GPIHBP1 gene identified in 2 patients with recurrent acute pancreatitis. J. Clin. Lipidol. 10, 92–100. 10.1016/j.jacl.2015.09.007 26892125

[B4] Ariza M. J. Rioja Villodres J. Ibarretxe D. Camacho A. Díaz-Díaz J. L. Mangas A. (2018). Molecular basis of the familial chylomicronemia syndrome in patients from the national dyslipidemia registry of the Spanish atherosclerosis society. J. Clin. Lipidol. 12, 1482–1492. e3. 10.1016/j.jacl.2018.07.013 30150141

[B5] Arora R. Nimonkar A. v. Baird D. Wang C. Chiu C. H. Horton P. A. (2019). Structure of lipoprotein lipase in complex with GPIHBP1. Proc. Natl. Acad. Sci. U. S. A. 116, 10360–10365. 10.1073/pnas.1820171116 31072929 PMC6534989

[B6] Basit A. Sabir S. Riaz M. Fawwad A. Abro M. U. R. Ahmed K. I. (2020). Ndsp 05: Prevalence and pattern of dyslipidemia in urban and rural areas of Pakistan; a sub analysis from second National Diabetes Survey of Pakistan (NDSP) 2016–2017. J. Diabetes Metab. Disord. 19, 1215–1225. 10.1007/s40200-020-00631-z 33520835 PMC7843689

[B7] Beigneux A. P. Fong L. G. Bensadoun A. Davies B. S. J. Oberer M. Gårdsvoll H. (2015). GPIHBP1 missense mutations often cause multimerization of GPIHBP1 and thereby prevent lipoprotein lipase binding. Circ. Res. 116, 624–632. 10.1161/CIRCRESAHA.116.305085 25387803 PMC4329087

[B8] Beigneux A. P. Franssen R. Bensadoun A. Gin P. Melford K. Peter J. (2009a). Chylomicronemia with a mutant GPIHBP1 (Q115P) that cannot bind lipoprotein lipase. Arterioscler. Thromb. Vasc. Biol. 29, 956–962. 10.1161/ATVBAHA.109.186577 19304573 PMC2811263

[B9] Beigneux A. P. Gin P. Davies B. S. J. Weinstein M. M. Bensadoun A. Fong L. G. (2009b). Highly conserved cysteines within the Ly6 domain of GPIHBP1 are crucial for the binding of lipoprotein lipase. J. Biol. Chem. 284, 30240–30247. 10.1074/jbc.M109.046391 19726683 PMC2781579

[B10] Beigneux A. P. Miyashita K. Ploug M. Blom D. J. Ai M. Linton M. F. (2017). Autoantibodies against GPIHBP1 as a cause of hypertriglyceridemia. N. Engl. J. Med. 376, 1647–1658. 10.1056/nejmoa1611930 28402248 PMC5555413

[B11] Beigneux A. R. Weinstein M. M. Davies B. S. J. Gin P. Bensadoun A. Fong L. G. (2009c). GPIHBP1 and lipolysis: An update. Curr. Opin. Lipidol. 20, 211–216. 10.1097/MOL.0b013e32832ac026 19369870 PMC2810420

[B12] Berge K. E. Retterstøl K. Romeo S. Pirazzi C. Leren T. P. (2014). Type 1 hyperlipoproteinemia due to a novel deletion of exons 3 and 4 in the GPIHBP1 gene. Atherosclerosis 234, 30–33. 10.1016/j.atherosclerosis.2014.02.005 24589565

[B13] Brown E. E. Sturm A. C. Cuchel M. Braun L. T. Duell P. B. Underberg J. A. (2020). Genetic testing in dyslipidemia: A scientific statement from the national lipid association. J. Clin. Lipidol. 14, 398–413. 10.1016/j.jacl.2020.04.011 32507592

[B14] Buonuomo P. S. Bartuli A. Rabacchi C. Bertolini S. Calandra S. (2015). A 3-day-old neonate with severe hypertriglyceridemia from novel mutations of the GPIHBP1 gene. J. Clin. Lipidol. 9, 265–270. 10.1016/j.jacl.2014.10.003 25911085

[B15] Carrasquilla G. D. Christiansen M. R. Kilpeläinen T. O. (2021). The genetic basis of hypertriglyceridemia. Curr. Atheroscler. Rep. 23, 39. 10.1007/s11883-021-00939-y 34146174 PMC8214584

[B16] Charrière S. Peretti N. Bernard S. di Filippo M. Sassolas A. Merlin M. (2011). GPIHBP1 C89F neomutation and hydrophobic C-terminal domain G175R mutation in two pedigrees with severe hyperchylomicronemia. J. Clin. Endocrinol. Metab. 96, 1675–1679. 10.1210/jc.2011-1444 21816778

[B17] Chokshi N. Blumenschein S. D. Ahmad Z. Garg A. (2014). Genotype-phenotype relationships in patients with type i hyperlipoproteinemia. J. Clin. Lipidol. 8, 287–295. 10.1016/j.jacl.2014.02.006 24793350

[B18] Chyzhyk V. Kozmic S. Brown A. S. Hudgins L. C. Starc T. J. Davila A. D. (2019). Extreme hypertriglyceridemia: Genetic diversity, pancreatitis, pregnancy, and prevalence. J. Clin. Lipidol. 13, 89–99. 10.1016/j.jacl.2018.09.007 30352774

[B19] Coca-Prieto I. Kroupa O. Gonzalez-Santos P. Magne J. Olivecrona G. Ehrenborg E. (2011). Childhood-onset chylomicronaemia with reduced plasma lipoprotein lipase activity and mass: Identification of a novel GPIHBP1 mutation. J. Intern. Med. 270, 224–228. 10.1111/j.1365-2796.2011.02361.x 21314738

[B20] Cruz-Bautista I. Huerta-Chagoya A. Moreno-Macías H. Rodríguez-Guillén R. Ordóñez-Sánchez M. L. Segura-Kato Y. (2021). Familial hypertriglyceridemia: An entity with distinguishable features from other causes of hypertriglyceridemia. Lipids Health Dis. 20, 14. 10.1186/s12944-021-01436-6 33588820 PMC7885394

[B21] De Jesus J. M. (2011). Expert panel on integrated guidelines for cardiovascular health and risk reduction in children and adolescents: Summary report. Pediatrics 128, S213–S256. 10.1542/peds.2009-2107C 22084329 PMC4536582

[B22] Desvignes J. Bartoli M. Krahn M. Miltgen M. Christophe B. Salgado D. (2018). VarAFT : A variant annotation and filtration system for human next generation sequencing data. Nucleic Acids Res. 46, W545–W553. 10.1093/nar/gky471 29860484 PMC6030844

[B23] Esan O. Wierzbicki A. S. (2020). Volanesorsen in the treatment of familial chylomicronemia syndrome or hypertriglyceridaemia: Design, development and place in therapy. Drug Des. devel. Ther. 14, 2623–2636. 10.2147/DDDT.S224771 PMC7351689 32753844

[B24] Fong L. G. Young S. G. Beigneux A. P. Bensadoun A. Oberer M. Jiang H. (2016). GPIHBP1 and plasma triglyceride metabolism. Trends Endocrinol. Metab. 27, 455–469. 10.1016/j.tem.2016.04.013 27185325 PMC4927088

[B25] Franklin by Genoox (2022). Available at: https://franklin.genoox.com (Accessed May 30, 2022).

[B26] Franssen R. Young S. G. Peelman F. Hertecant J. Sierts J. A. Schimmel A. W. M. (2010). Chylomicronemia with low postheparin lipoprotein lipase levels in the setting of GPIHBP1 defects. Circ. Cardiovasc. Genet. 3, 169–178. 10.1161/CIRCGENETICS.109.908905 20124439 PMC2858258

[B27] Gin P. Goulbourne C. N. Adeyo O. Beigneux A. P. Davies B. S. J. Tat S. (2012). Chylomicronemia mutations yield new insights into interactions between lipoprotein lipase and GPIHBP1. Hum. Mol. Genet. 21, 2961–2972. 10.1093/hmg/dds127 22493000 PMC3373243

[B28] Goldberg A. S. Hegele R. A. (2012). Severe hypertriglyceridemia in pregnancy. J. Clin. Endocrinol. Metab. 97, 2589–2596. 10.1210/jc.2012-1250 22639290

[B29] Goldberg R. B. Chait A. (2020). A comprehensive update on the chylomicronemia syndrome. Front. Endocrinol. 11, 593931. 10.3389/fendo.2020.593931 PMC7644836 33193106

[B30] Gonzaga-Jauregui C. Mir S. Penney S. Jhangiani S. Midgen C. Finegold M. (2014). Whole-exome sequencing reveals GPIHBP1 mutations in infantile colitis with severe hypertriglyceridemia. J. Pediatr. Gastroenterol. Nutr. 59, 17–21. 10.1097/MPG.0000000000000363 24614124 PMC4203304

[B31] Groselj U. Kovac J. Sustar U. Mlinaric M. Fras Z. Podkrajsek K. T. (2018). Universal screening for familial hypercholesterolemia in children: The Slovenian model and literature review. Atherosclerosis 277, 383–391. 10.1016/j.atherosclerosis.2018.06.858 30270075

[B32] Groselj U. Wiegman A. Gidding S. S. (2022). Screening in children for familial hypercholesterolaemia: Start now. Eur. Heart J., ehac224. 10.1093/eurheartj/ehac224 35511818

[B33] Hegele R. A. Berberich A. J. Ban M. R. Wang J. Digenio A. Alexander V. J. (2018). Clinical and biochemical features of different molecular etiologies of familial chylomicronemia. J. Clin. Lipidol. 12, 920–927. e4. 10.1016/j.jacl.2018.03.093 29748148

[B34] Hegele R. A. Borén J. Ginsberg H. N. Arca M. Averna M. Binder C. J. (2020). Rare dyslipidaemias, from phenotype to genotype to management: A European atherosclerosis society task force consensus statement. Lancet. Diabetes Endocrinol. 8, 50–67. 10.1016/S2213-8587(19)30264-5 31582260

[B35] Holmes R. S. Cox L. A. (2012). Comparative studies of glycosylphosphatidylinositol-anchored high-density lipoprotein-binding protein 1: Evidence for a eutherian mammalian origin for the GPIHBP1 gene from an LY6-like gene. 3 Biotech. 2, 37–52. 10.1007/s13205-011-0026-4 PMC3339605 22582156

[B36] Iacocca M. A. Dron J. S. Hegele R. A. (2019). Progress in finding pathogenic DNA copy number variations in dyslipidemia. Curr. Opin. Lipidol. 30, 63–70. 10.1097/MOL.0000000000000581 30664016

[B37] Ioannidis N. M. Rothstein J. H. Pejaver V. Middha S. McDonnell S. K. Baheti S. (2016). Revel: An ensemble method for predicting the pathogenicity of rare missense variants. Am. J. Hum. Genet. 99, 877–885. 10.1016/j.ajhg.2016.08.016 27666373 PMC5065685

[B38] Jung M. K. Jin J. Kim H. O. Kwon A. Chae H. W. Kang S. J. (2017). A 1-month-old infant with chylomicronemia due to GPIHBP1 gene mutation treated by plasmapheresis. Ann. Pediatr. Endocrinol. Metab. 22, 68–71. 10.6065/apem.2017.22.1.68 28443263 PMC5401827

[B39] Karczewski K. J. Francioli L. C. Tiao G. Cummings B. B. Alföldi J. Wang Q. (2020). The mutational constraint spectrum quantified from variation in 141, 456 humans. Nature 581, 434–443. 10.1038/s41586-020-2308-7 32461654 PMC7334197

[B40] Kersten S. (2021). Role and mechanism of the action of angiopoietin-like protein ANGPTL4 in plasma lipid metabolism. J. Lipid Res. 62, 100150. 10.1016/J.JLR.2021.100150 34801488 PMC8666355

[B41] Landrum M. J. Lee J. M. Benson M. Brown G. R. Chao C. Chitipiralla S. (2018). ClinVar: Improving access to variant interpretations and supporting evidence. Nucleic Acids Res. 46, D1062–D1067. 10.1093/nar/gkx1153 29165669 PMC5753237

[B42] Lima J. G. Helena C Nobrega L. Moura Bandeira F. T. Pires Sousa A. G. Medeiros de Araujo Macedo T. B. Cavalcante Nogueira A. C. (2021). A novel GPIHBP1 mutation related to familial chylomicronemia syndrome: A series of cases. Atherosclerosis 322, 31–38. 10.1016/j.atherosclerosis.2021.02.020 33706081

[B43] Lin M. H. Tian X. H. Hao X. L. Fei H. Yin J. L. Yan D. D. (2020). Management of a pregnant patient with chylomicronemia from a novel mutation in GPIHBP1: A case report. BMC Pregnancy Childbirth 20, 272. 10.1186/s12884-020-02965-1 32375710 PMC7201967

[B44] Liu C. Li L. Guo D. Lv Y. Zheng X. L. Mo Z. (2018). Lipoprotein lipase transporter GPIHBP1 and triglyceride-rich lipoprotein metabolism. Clin. Chim. Acta. 487, 33–40. 10.1016/j.cca.2018.09.020 30218660

[B45] Liu S. Wang Z. Zheng X. Zhang Y. Wei S. OuYang H. (2022). Case report: Successful management of a 29-day-old infant with severe hyperlipidemia from a novel homozygous variant of GPIHBP1 gene. Front. Pediatr. 10, 792574. 10.3389/fped.2022.792574 35359903 PMC8960264

[B46] Marusic T. Sustar U. Sadiq F. Kotori V. Mlinaric M. Kovac J. (2020). Genetic and clinical characteristics of patients with homozygous and compound heterozygous familial hypercholesterolemia from three different populations: Case series. Front. Genet. 11, 572176. 10.3389/fgene.2020.572176 33093846 PMC7528874

[B47] Miyashita K. Lutz J. Hudgins L. C. Toib D. Ashraf A. P. Song W. (2020). Chylomicronemia from GPIHBP1 autoantibodies. J. Lipid Res. 61, 1365–1376. 10.1194/jlr.R120001116 32948662 PMC7604722

[B48] Mysling S. Kristensen K. K. Larsson M. Beigneux A. P. Gårdsvoll H. Loren F. G. (2016). The acidic domain of the endothelial membrane protein GPIHBP1 stabilizes lipoprotein lipase activity by preventing unfolding of its catalytic domain. Elife 5, e12095. 10.7554/eLife.12095 26725083 PMC4755760

[B49] Navarro Hermoso A. Valdivielso P. (2021). Treatment of chylomicronemia. Clin. Investig. Arterioscler. 33, 75–79. 10.1016/j.arteri.2021.01.004 34006359

[B50] Nordestgaard B. G. (2016). Triglyceride-rich lipoproteins and atherosclerotic cardiovascular disease: New insights from epidemiology, genetics, and biology. Circ. Res. 118, 547–563. 10.1161/CIRCRESAHA.115.306249 26892957

[B51] Okazaki H. Gotoda T. Ogura M. Ishibashi S. Inagaki K. Daida H. (2021). Current diagnosis and management of primary chylomicronemia. J. Atheroscler. Thromb. 28, 883–904. 10.5551/jat.RV17054 33980761 PMC8532063

[B52] Olivecrona G. Ehrenborg E. Semb H. Makoveichuk E. Lindberg A. Hayden M. R. (2010). Mutation of conserved cysteines in the Ly6 domain of GPIHBP1 in familial chylomicronemia. J. Lipid Res. 51, 1535–1545. 10.1194/jlr.M002717 20026666 PMC3035517

[B53] Paquette M. Hegele R. A. Paré G. Baass A. (2018). A novel mutation in GPIHBP1 causes familial chylomicronemia syndrome. J. Clin. Lipidol. 12, 506–510. 10.1016/j.jacl.2018.01.011 29452893

[B54] Parhofer K. G. Laufs U. (2019). The diagnosis and treatment of hypertriglyceridemia. Dtsch. Arztebl. Int. 116, 825–832. 10.3238/arztebl.2019.0825 31888796 PMC6962767

[B55] Patni N. Brothers J. Xing C. Garg A. (2016). Type 1 hyperlipoproteinemia in a child with large homozygous deletion encompassing GPIHBP1. J. Clin. Lipidol. 10, 1035–1039. e2. 10.1016/j.jacl.2016.04.001 27578137

[B56] Plengpanich W. Young S. G. Khovidhunkit W. Bensadoun A. Karnman H. Ploug M. (2014). Multimerization of glycosylphosphatidylinositol-anchored high density lipoprotein-binding protein 1 (GPIHBP1) and familial chylomicronemia from a serine-to-cysteine substitution in GPIHBP1 Ly6 domain. J. Biol. Chem. 289, 19491–19499. 10.1074/jbc.M114.558528 24847059 PMC4094059

[B57] Rabacchi C. D’Addato S. Palmisano S. Lucchi T. Bertolini S. Calandra S. (2016). Clinical and genetic features of 3 patients with familial chylomicronemia due to mutations in GPIHBP1 gene. J. Clin. Lipidol. 10, 915–921. e4. 10.1016/j.jacl.2016.03.009 27578123

[B58] Remec Z. I. Trebusak Podkrajsek K. Repic Lampret B. Kovac J. Groselj U. Tesovnik T. (2021). Next-generation sequencing in newborn screening: A review of current state. Front. Genet. 12, 662254. 10.3389/fgene.2021.662254 34122514 PMC8188483

[B59] Richards S. Aziz N. Bale S. Bick D. Das S. Gastier-Foster J. (2015). Standards and guidelines for the interpretation of sequence variants: A joint consensus recommendation of the American College of medical genetics and genomics and the association for molecular Pathology. Genet. Med. 17, 405–424. 10.1038/gim.2015.30 25741868 PMC4544753

[B60] Rios J. J. Shastry S. Jasso J. Hauser N. Garg A. Bensadoun A. (2012). Deletion of GPIHBP1 causing severe chylomicronemia. J. Inherit. Metab. Dis. 35, 531–540. 10.1007/s10545-011-9406-5 22008945 PMC3319888

[B61] Silverman M. G. Ference B. A. Im K. Wiviott S. D. Giugliano R. P. Grundy S. M. (2016). Association between lowering ldl-C and cardiovascular risk reduction among different therapeutic interventions: A systematic review and meta-analysis. JAMA - J. Am. Med. Assoc. 316, 1289–1297. 10.1001/jama.2016.13985 27673306

[B62] Simha V. (2020). Management of hypertriglyceridemia. BMJ 371, m3109. 10.1136/bmj.m3109 33046451

[B63] Stenson P. D. Ball E. v. Mort M. Phillips A. D. Shiel J. A. Thomas N. S. T. (2003). Human gene mutation database (HGMD): 2003 update. Hum. Mutat. 21, 577–581. 10.1002/humu.10212 12754702

[B64] Surendran R. P. Visser M. E. Heemelaar S. Wang J. Peter J. Defesche J. C. (2012). Mutations in LPL, APOC2, APOA5, GPIHBP1 and LMF1 in patients with severe hypertriglyceridaemia. J. Intern. Med. 272, 185–196. 10.1111/j.1365-2796.2012.02516.x 22239554 PMC3940136

[B65] Sustar U. Groselj U. Trebusak Podkrajsek K. Mlinaric M. Kovac J. Thaler M. (2022). Early discovery of children with lysosomal acid lipase deficiency with the universal familial hypercholesterolemia screening program. Front. Genet. IN PRESS. 10.3389/fgene.2022.936121 PMC9314654 35903350

[B66] Tromp T. R. Hartgers M. L. Hovingh G. K. Vallejo-Vaz A. J. Ray K. K. Soran H. (2022). Worldwide experience of homozygous familial hypercholesterolaemia: Retrospective cohort study. Lancet 399, 719–728. 10.1016/S0140-6736(21)02001-8 35101175 PMC10544712

[B67] UniProt (2022). UniProt: The universal protein knowledgebase in 2021. Nucleic Acids Res. 49 (D1), D480–D489. Available at: https://www.uniprot.org/uniprot/Q8IV16#family_and_domains (Accessed May 2, 2022). 10.1093/nar/gkaa11008 PMC7778908 33237286

[B68] Vallejo Vaz A. J. Stevens C. A. T. Lyons A. R. M. Dharmayat K. I. Freiberger T. Hovingh G. K. (2021). Global perspective of familial hypercholesterolaemia : A cross-sectional study from the EAS familial hypercholesterolaemia studies collaboration ( FHSC ). Lancet 6736, 1713–1725. 10.1016/S0140-6736(21)01122-3 34506743

[B69] Vallejo-Vaz A. J. Marco M. De Stevens C. A. T. Akram A. Freiberger T. Hovingh G. K. (2018). Overview of the current status of familial hypercholesterolaemia care in over 60 countries - the EAS Familial Hypercholesterolaemia Studies Collaboration (FHSC). Atherosclerosis 277, 234–255. 10.1016/j.atherosclerosis.2018.08.051 30270054

[B70] Voss C. v. Davies B. S. J. Tat S. Gin P. Fong L. G. Pelletier C. (2011). Mutations in lipoprotein lipase that block binding to the endothelial cell transporter GPIHBP1. Proc. Natl. Acad. Sci. U. S. A. 108, 7980–7984. 10.1073/pnas.1100992108 21518912 PMC3093490

[B71] Wang J. Hegele R. A. (2007). Homozygous missense mutation (G56R) in glycosylphosphatidylinositol- anchored high-density lipoprotein-binding protein 1 (GPI-HBP1) in two siblings with fasting chylomicronemia (MIM 144650). Lipids Health Dis. 6, 23–27. 10.1186/1476-511X-6-23 17883852 PMC2039732

[B72] Wu S. A. Kersten S. Qi L. (2021). Lipoprotein lipase and its regulators: An unfolding story. Trends Endocrinol. Metab. 32, 48–61. 10.1016/j.tem.2020.11.005 33277156 PMC8627828

[B73] Yamamoto H. Onishi M. Miyamoto N. Oki R. Ueda H. Ishigami M. (2013). Novel combined GPIHBP1 mutations in a patient with hypertriglyceridemia associated with CAD. J. Atheroscler. Thromb. 20, 777–784. 10.5551/jat.18861 23831619

