# Supplementary material for: A homozygous variant in the GPIHBP1 gene in a child with severe hypertriglyceridemia and a systematic literature review
Source: Front Genet. 2022 Aug 16;13:983283. doi: 10.3389/fgene.2022.983283 (PMC9424485; doi:10.3389/fgene.2022.983283)
Supplement: Supplementary file 1 [file Presentation1.pdf]

Supplementary Material

Supplementary Figures

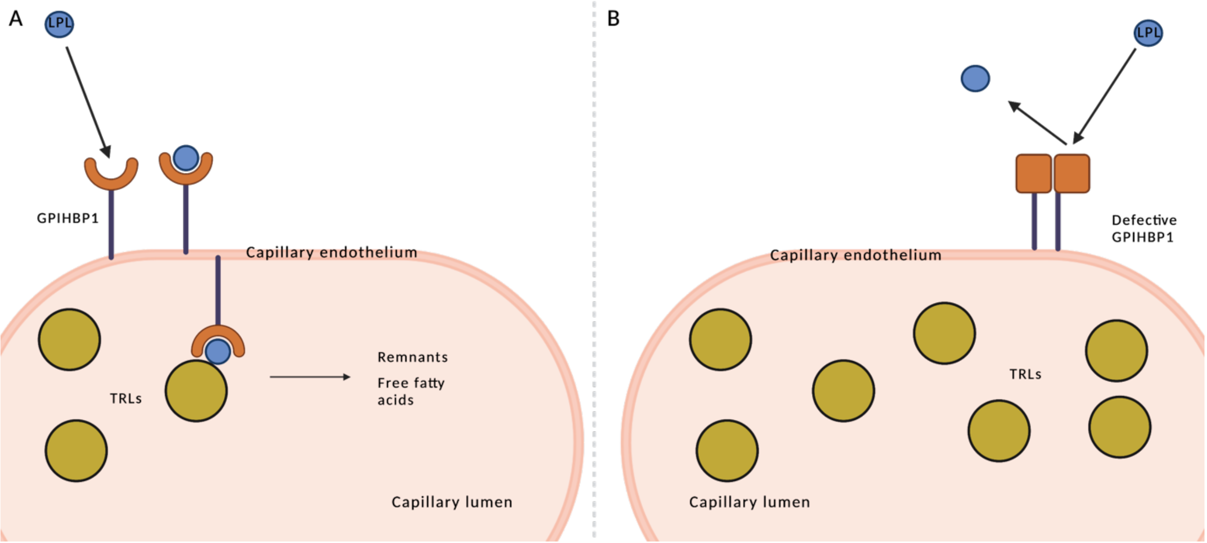

Supplementary Figure 1: Mechanism of hydrolysis of triglycerides packed in the lipoproteins

(A) GPIHBP1 binds to LPL and transports it from the interstitial space to the capillary lumen, where it hydrolyses triglycerides (TG) and triglyceride-rich lipoproteins (TRLs) to free fatty acids and remnants. (B) When GPIHBP1 is defective due to a pathogenic variant in the *GPIHBP1* gene, LPL is not transported at the site of its action and hydrolysis of TG and TRLs is impaired, leading to severe hypertriglyceridemia.

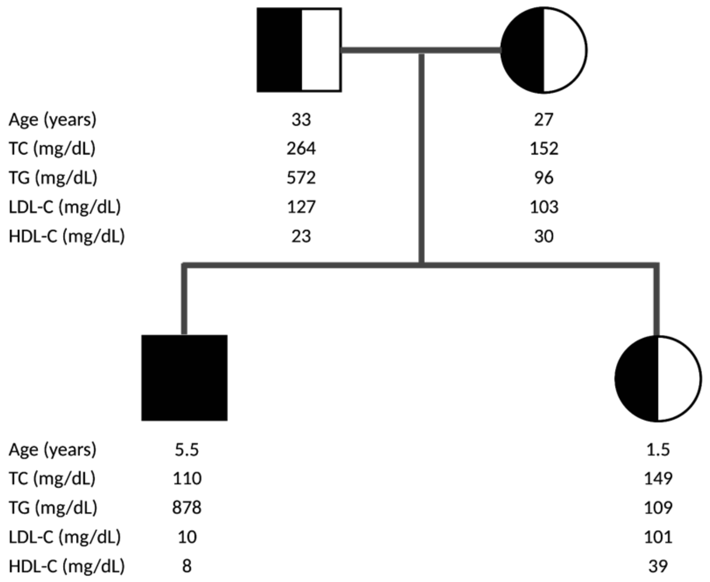

Supplementary Figure 2: Pedigree of the patient's family

Lipid profile and age are represented at the time of the genetic testing of the family. The filled symbol indicated the affected proband. The half-filled symbol indicates heterozygous carriers

of the variant in the *GPIHBP1* gene. At the time of genetic diagnosis and lipidogram measurement, the patient was treated with fenofibrate and gemfibrozil.

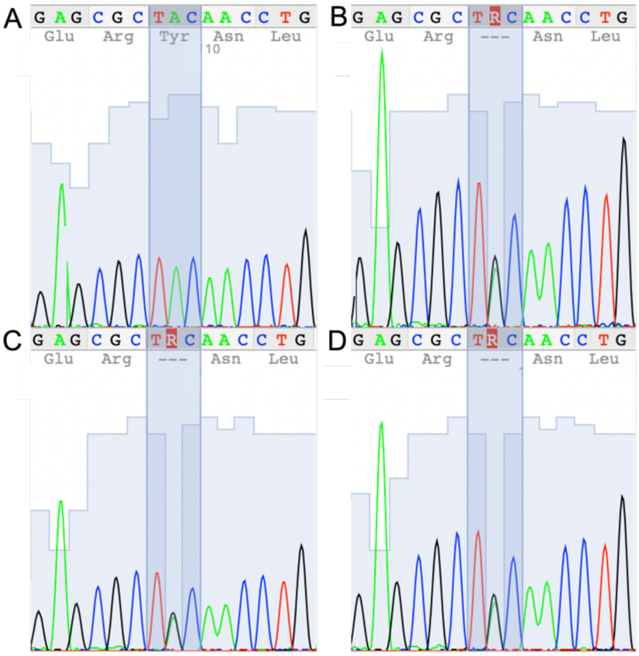

Supplementary Figure 3: Chromatograms as a confirmatory analysis of the patient with homozygous and his family members with heterozygous variant c.230G>A (p.Cys77Tyr) in the *GPIHBP1* gene

Homozygous variant c.230G>A (NM_178172.6) in exon 3 of the *GPIHBP1* gene was reconfirmed by the targeted Sanger sequencing method. Amino acid cysteine (TGC) at position 77 was replaced by amino acid tyrosine (TAC). Chromatograms of the (A) homozygous patient, (B) heterozygous patient's father, (C) heterozygous patient's mother, and (D) heterozygous patient's sister.

Table 1: Lipoprotein and glucose levels of the patient over time

Age	(years)	0.9	1.7	1.9	2.0	2.9	3.3	4.5	4.9	5.6	5.9
**TC**	**(mg/dL)**	850.7	368	636	370	81	118	263	158	110	74
	**(mmol/L)**	22.0	9.5	16.4	9.6	2.1	3.1	6.8	4.1	2.8	1.9
**TG**	**(mg/dL)**	5137			3720	811	1039	2883	3749	878	630
	**(mmol/L)**	58.0			42.0	9.2	11.7	32.6	42.3	9.9	7.1
**LDL-C**	**(mg/dL)**					18.0	16.0	9.0	9.0	10.0	10.0
	**(mmol/L)**					0.5	0.4	0.2	0.2	0.3	0.3
**HDL**	**(mg/dL)**					8.0	10.0	10.0	6.0	8.0	8.0
	**(mmol/L)**					0.2	0.3	0.3	0.2	0.2	0.2
**Glucose**	**(mg/dL)**	29.5			180.0						
	**(mmol/L)**	1.6			10.0						
**Bilirubin**	**(mg/dL)**	6.4									
	**(umol/L)**	110.0									

TC: total cholesterol; TG: triglycerides; LDL-C: low-density lipoprotein cholesterol; HDL: high-density lipoprotein cholesterol
